# Meigs syndrome after treatment for catamenial pneumothorax

**DOI:** 10.1186/s44215-023-00110-w

**Published:** 2023-11-01

**Authors:** Mao Takayama, Tetsuya So, Naoki Yamashita, Masatoshi Yamaoka, Takashi Yoshimatsu, Tsuneiro Oyama

**Affiliations:** 1 Department of Thoracic Surgery, Shinkomonji Hospital, 2-5 Dairishinmachi, Moji-Ku, Kitakyushu, 800-0057 Japan; 2 Department of Thoracic Surgery, Fukuoka-Wajiro Hospital, 2-2-75 Wajirogaoka, Higashi-Ku, Fukuoka, 811-0213 Japan; 3 Department of Surgery, Imamitsu Homecare Clinic, 1-9-10 Imamitsu, Wakamatsu-Ku, Kitakyushu, 808-0071 Japan

**Keywords:** Meigs syndrome, Catamenial pneumothorax, Ovarian tumors

## Abstract

In the field of thoracic surgery, catamenial pneumothorax (CP) is known as a disease peculiar to women, but it is rare among female pneumothoraces and is rarely encountered in clinical practice. Meigs syndrome (MS) is another female-specific disease, defined as a benign ovarian tumor with pleural and ascites effusions, but it is rare and the details of the pathogenesis of MS have not yet been elucidated.

A 40-year-old Japanese woman came in with dyspnea. Chest radiography revealed a collapsed right lung. She underwent video-assisted thoracoscopic surgery, which revealed multiple diaphragmatic foramens. She was therefore diagnosed with CP. Later, when she was 50-year-old, returned with chest pain. Computed tomography of the chest and abdomen showed right massive pleural effusion and a large tumor and ascites in the pelvis. This condition was suggestive of MS. The patient underwent bilateral oophorectomy, and the right pleural effusion and ascites resolved promptly after surgery.

In conclusion, both menstrual-associated CP and MS are very rare conditions, and to the best of our knowledge, there are no reported cases of the combination of the two. However, it is possible that some MS patients may have MS without CP, and the present case is considered to be valuable because a small diaphragmatic foramen was identified via thoracoscopy, which has been a minority opinion among the mechanisms of pleural effusion in MS.

## Introduction

Catamenial pneumothorax (CP) is known in the field of thoracic surgery as a disease peculiar to women. CP is the most common manifestation of thoracic endometriosis. Because CP is a rare disease, it is rarely encountered in clinical practice. Conversely, Meigs syndrome (MS) is also a disease specific to women and is defined as a benign ovarian tumor with pleural effusion and ascites. MS is also a rare disease, and the details of the mechanism underlying MS have not yet been elucidated. Herein, we report an extremely rare case of CP followed by MS.

## Case report

**Fig. 1:**
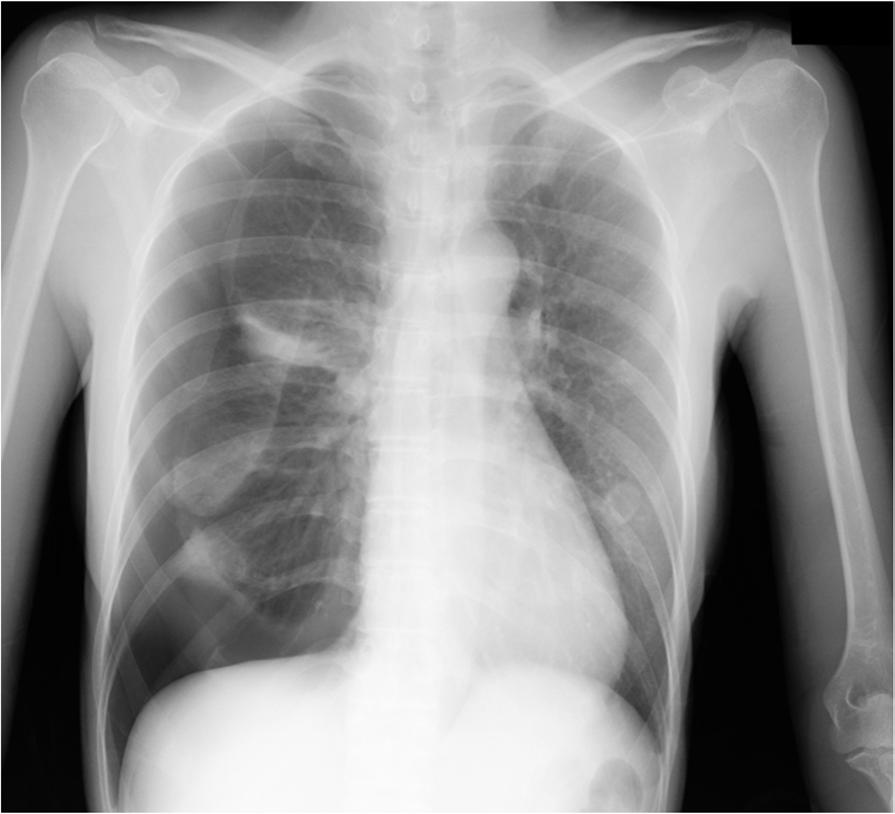
Chest radiography showing right pneumothorax

**Fig. 2:**
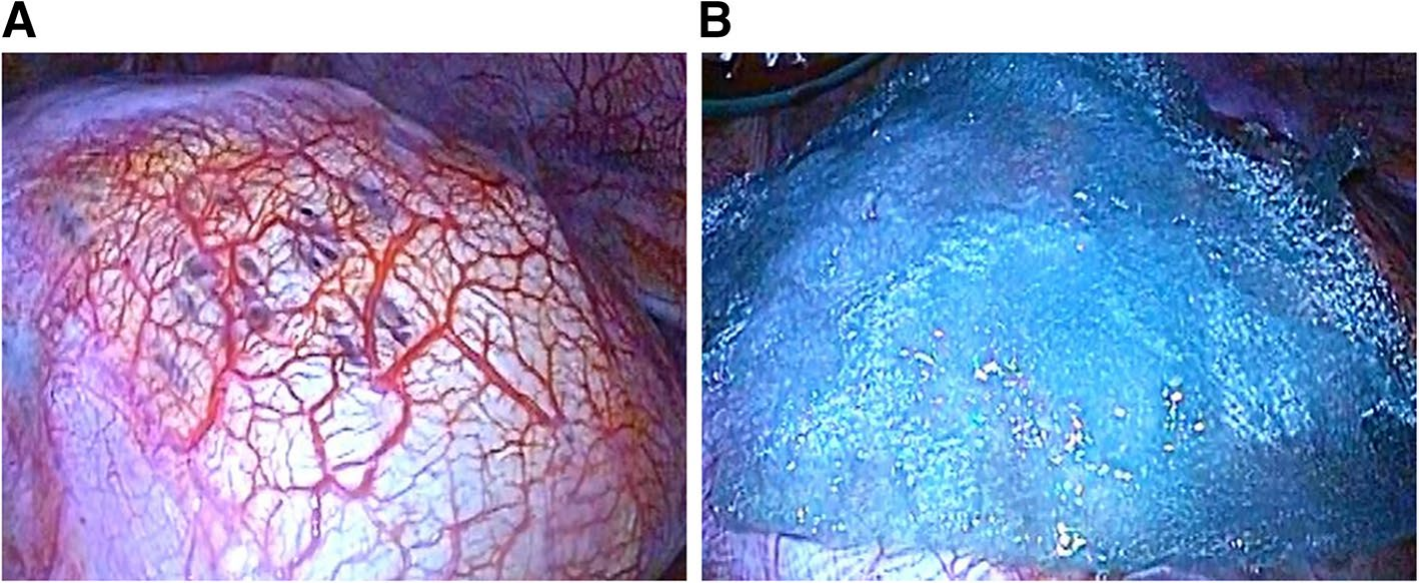
Surgical findings. **A** Multiple diaphragmatic foramens. **B** A PGA sheet was applied to the entire diaphragm

A 40-year-old Japanese woman presented to our hospital (Shinkomonji Hospital, Kitakyushu, Japan) due to dyspnea and right-sided chest pain. These symptoms occurred within 48 h after the onset of menstruation. Chest radiography revealed a collapsed right lung (Fig. 1). Chest computed tomography (CT) showed no pleural effusion and no emphysematous changes or neoplastic lesions (data not shown); thus, CP was suggested. Although we performed right thoracic drainage, minor air leakage continued. The patient underwent video-assisted thoracoscopic surgery. Thoracoscopy revealed multiple diaphragmatic foramens around the central tendon (Fig. 2A); however, no abnormal legions were noted at the visceral pleura or wall-side pleura. Intraoperative water sealing test showed no obvious leak and no partial excision was performed. It is difficult to perform complete thoracoscopic resection of the diaphragm, and hormone therapy is fundamental to this disease, and surgery is not performed in consideration of the degree of invasiveness. The entire surface of the diaphragm was covered with a polyglycolic acid (PGA) sheet (Fig. 2B) and fibrin glue.

As a result of consultation with an obstetrician and gynecologist using the clinical course and intraoperative findings, the patient was thus diagnosed with CP and underwent hormonal therapy with danazol. No clinical recurrence of CP was noted thereafter.

**Fig. 3:**
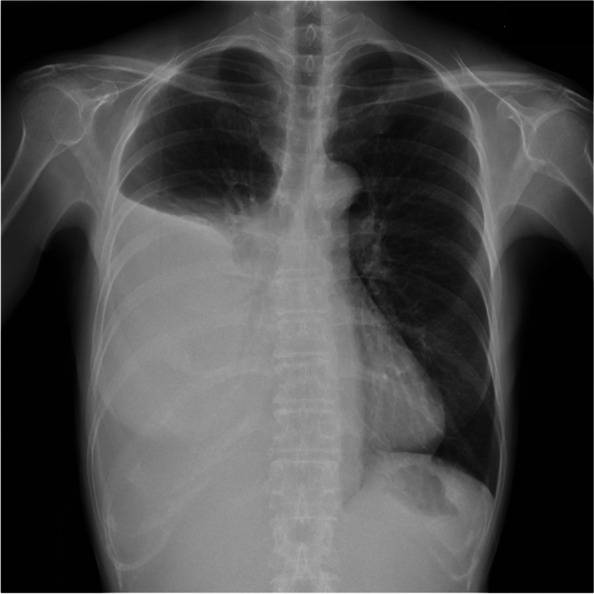
Chest radiography revealed a right massive pleural effusion

**Fig. 4:**
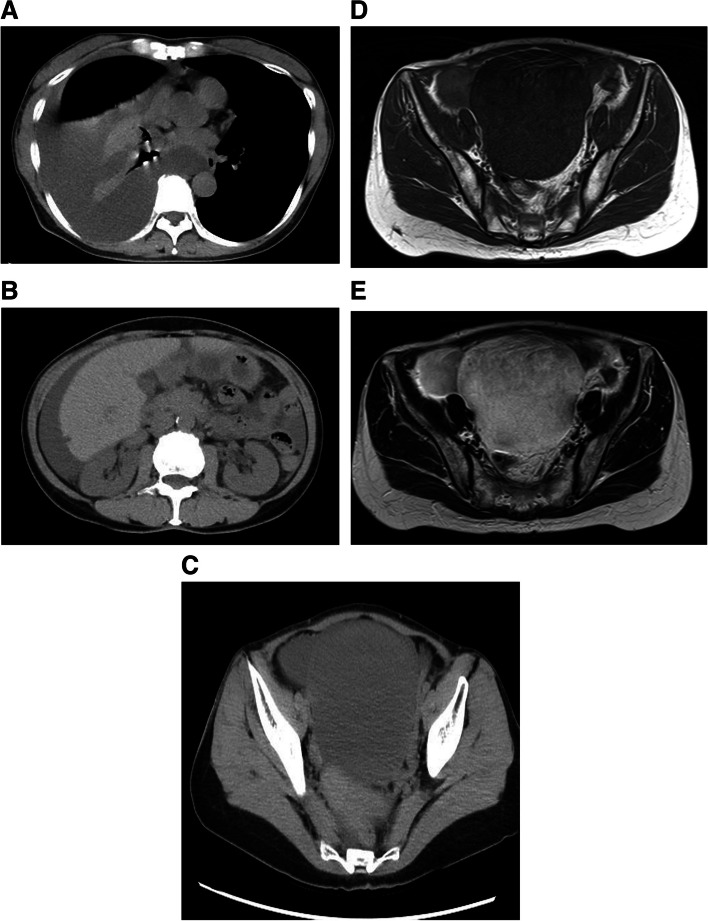
CT. **A** Chest: pleural effusion, passive atelectasis. **B**, **C** Abdomen and pelvis: ascites and a huge tumor 10 cm in diameter in the pelvis. MRI. **D** Hypointense signals on T1-weighted MRI. **E** Hyperintense signals on T2-weighted MRI

Ten years later (at the age of 50 years), the patient returned to our hospital with a complaint of chest pain. Chest radiography revealed a right massive pleural effusion (Fig. 3). Chest CT showed pleural effusion and passive atelectasis (Fig. 4A). Abdominal CT detected ascites (Fig. 4B) and a huge tumor with a regular margin measuring 10 × 9 cm in size at her pelvis (Fig. 4C). Abdominal magnetic resonance imaging (MRI) revealed hypointense signals on T1-weighted MRI (Fig. 4D) and hyperintense signals on T2-weighted MRI (Fig. 4E). The carbohydrate antigen 125 level was 645 U/mL (reference range: 0–35 U/mL). The combination of symptoms, including right pleural effusion, ascites, and ovarian tumor, led to the suspicion of MS. Therefore, we referred the patient to the Department of Gynecology, where gynecologists performed bilateral tubal oophorectomy. Histopathological examination of the resected tumors confirmed ovarian fibroma and no evidence of malignancy. After gynecologic surgery, a rapid disappearance of right pleural effusion and ascites was noted. The clinical course also suggested MS.

## Discussion

CP was first reported by Maurer in 1958 and remains a relatively rare disease [1]. The diagnostic criteria for CP is its appearance before or within 72 h after the start of monthly bleeding. The broad definition of CP is a pneumothorax occurrence from 7 days before the start of monthly bleeding to 7 days after the end of bleeding. Additional criteria include characteristic pleural lesions, right-sided location of the pneumothorax, and concomitant endometriosis.

Researchers have proposed three theories for the pathogenesis of CP. First, Mauer proposed a mechanism in which endometrial tissue is seeded into the thoracic cavity via a small foramen in the diaphragm and grows on the visceral pleura, resulting in pneumothorax with menstruation [2]. He also suggested that after endometrial tissue is deposited on the diaphragm, its shedding during menstruation forms a small foramen, resulting in pneumothorax. Second, Lillington reported that endometrial tissue migrates hematogenously into the peripheral airways, forming a bulla or bleb by the check valve mechanism, resulting in pneumothorax [3]. Third, Rossi suggested that high levels of circulating prostaglandin F2a, resulting from a collapsed endometrium, cause vasoconstriction and bronchospasm, leading to the rupture of the alveolar tissue [4].

Mauer’s theory is supported by the identification of endometriosis in the diaphragm in most patients with pneumothorax. However, these theories alone cannot explain all cases of CP without foramens in the diaphragm. Such cases may be caused by the mechanism due to Lillington and/or Rossi’s theory. In this case, small holes in the diaphragm were observed by thoracoscopic surgery 10 years previously.

MS was first reported by Meigs in 1937, who examined a series of seven patients with ascites and pleural effusion associated with benign ovarian fibroma [5]. Subsequently, this condition was defined as MS, with the following four characteristics: (1) benign ovarian fibroma or fibroma-like tumor, (2) ascites, (3) pleural effusion, and (4) rapid resolution of ascites and pleural effusion after tumor removal [6]. The pathogenesis of ascites and pleural effusion in MS remains unknown. Hamilton described that ascites may result from edematous fibromas that leak fluid or the increased lymphangial pressure in the abdomen and pelvis caused by the tumor itself. The lymphatics are abundant on the right side of the diaphragm, and pleural effusion is often observed on the right side [7]. Conversely, Okuda suggested that pleural effusion arises when ascites moves from the peritoneal cavity to the pleural cavity through diaphragmatic defects [8]. The latter theory is supported by the valuable case presented herein, in which small holes in the diaphragm were identified during the CP operation 10 years previously.

Despite robust hormonal therapy for CP, the patient developed MS. In this case, the diaphragm was fully covered with a PGA sheet for small holes in the diaphragm at the time of the CP operation. According to the instruction manual, a PGA sheet is almost completely absorbed in approximately 15 weeks. At the time of the CP operation, we believed that CP was a systemic gynecologic disease that was treated mainly via hormonal therapy. Furthermore, total resection and reconstruction of the diaphragm were highly invasive. Therefore, we decided to place the PGA sheet on the diaphragm for coverage. Murakami reported a case of artificial pericardial reinforcement using an expanded polytetrafluoroethylene (ePTFE) sheet for diaphragmatic hernia after CP diaphragmatic excision [9]. Because the CP diaphragm is very fragile, ePTFE sheets are considered to provide secure closure with sufficient strength. Presently, the most suitable surgical procedure for CP appears to be the total replacement of the diaphragm using the ePTFE sheet.

In this way, this patient developed MS unexpectedly, and the present case confirms the hypothesis of right pleural effusion in MS. Because endometriosis is a common gynecologic disease and is said to be increasing in recent years, it is possible that CP in a patient with endometriosis was mixed in with MS, in which no endometriosis was noted. Through this study, we believe that regular gynecological examinations are necessary for CP patients.

In conclusion, both menstrual-associated CP and MS are very rare conditions, and to the best of our knowledge, there are no reported cases of the combination of the two. The present case is considered to be valuable because a small diaphragmatic foramen was identified via thoracoscopy, which has been a minority opinion among the mechanisms of pleural effusion in MS.

## Data Availability

Not applicable.

## Abbreviations

CP: Catamenial pneumothorax
CT: Computed tomography
MS: Meigs syndrome
PGA: Polyglycolic acid
MRI: Magnetic resonance imaging
ePTFE: Expanded polytetrafluoroethylene

## References

[1] Marjański T. Catamenial pneumothorax – a review of the literature. Thorac Surg. 2016;13(2):117–21. 10.5114/kitp.2016.61044 PMC4971265 27516783

[2] Maurer ER, Schaal JA, Mendez FL. Chronic recurring spontaneous pneumothorax due to endometriosis of the diaphragm. J Am Med Assoc. 1958;168:2013–4. 13598643 10.1001/jama.1958.63000150008012c

[3] Lillington GA, Mitchell SP, Wood GA. Catamenial pneumothorax. JAMA. 1972;219:1328–32. 5066776

[4] Rossi NP, Goplerud CP. Recurrent catamenial pneumothorax. Arch Surg. 1974;109:173–6. 4846435 10.1001/archsurg.1974.01360020035008

[5] Corsellis JAN, Goldberg GJ, Norton AR. “Limbic encephalitis” and its association with carcinoma. Brain. 1968;91:481–96. 5723018 10.1093/brain/91.3.481

[6] Meigs JV, Cass JW. Fibroma of the ovary with ascites and hydrothorax with a report seven cases. Am J Obstet Gynecol. 1937;33:249–67.

[7] Okuda K. A case of Meigs’ syndrome, with special reference to the mechanism of pleural effusion. Int Med. 1967;20:569. 5588398

[8] Meigs JV. Fibroma of the ovary with ascites and hydrothorax Meigs’ syndrome. Am J Obstet Gynecol. 1954;67:962–85. 13148256 10.1016/0002-9378(54)90258-6

[9] Murakami T, et al. Post-operative diaphragmatic hernia in a patient with catamenial pneumothrax. J Clin Surg. 2011;66(5):701–4.

